# Failure of Decidualization and Maternal Immune Tolerance Underlies Uterovascular Resistance in Intra Uterine Growth Restriction

**DOI:** 10.3389/fendo.2019.00160

**Published:** 2019-03-20

**Authors:** Caroline Dunk, Melissa Kwan, Aleah Hazan, Sierra Walker, Julie K. Wright, Lynda K. Harris, Rebecca Lee Jones, Sarah Keating, John C. P. Kingdom, Wendy Whittle, Cynthia Maxwell, Stephen J. Lye

**Affiliations:** ^1^Research Centre for Women's and Infants' Health, Lunenfeld Tanenbaum Research Institute, Mount Sinai Hospital, Sinai Health System, Toronto, ON, Canada; ^2^Division of Pharmacy and Optometry, University of Manchester, Manchester, United Kingdom; ^3^Faculty of Biology Medicine and Health, Maternal and Fetal Health Research Centre, University of Manchester, Manchester, United Kingdom; ^4^Academic Health Science Centre, St Mary's Hospital, Manchester, United Kingdom; ^5^Department of Obstetrics and Gynaecology, Faculty of Medicine, University of Toronto, Toronto, ON, Canada; ^6^Department of Physiology, Faculty of Medicine, University of Toronto, Toronto, ON, Canada

**Keywords:** IUGR, uterovascular transformation, decidua, EVT, immunology, T cells, dendritic, progesterone

## Abstract

Failure of uterine vascular transformation is associated with pregnancy complications including Intra Uterine Growth Restriction (IUGR). The decidua and its immune cell populations play a key role in the earliest stages of this process. Here we investigate the hypothesis that abnormal decidualization and failure of maternal immune tolerance in the second trimester may underlie the uteroplacental pathology of IUGR. Placental bed biopsies were obtained from women undergoing elective caesarian delivery of a healthy term pregnancy, an IUGR pregnancy or a pregnancy complicated by both IUGR and preeclampsia. Decidual tissues were also collected from second trimester terminations from women with either normal or high uterine artery Doppler pulsatile index (PI). Immunohistochemical image analysis and flow cytometry were used to quantify vascular remodeling, decidual leukocytes and decidual status in cases vs. controls. Biopsies from pregnancies complicated by severe IUGR with a high uterine artery pulsatile index (PI) displayed a lack of: myometrial vascular transformation, interstitial, and endovascular extravillous trophoblast (EVT) invasion, and a lower number of maternal leukocytes. Apoptotic mural EVT were observed in association with mature dendritic cells and T cells in the IUGR samples. Second trimester pregnancies with high uterine artery PI displayed a higher incidence of small for gestational age fetuses; a skewed decidual immunology with higher numbers of; CD8 T cells, mature CD83 dendritic cells and lymphatic vessels that were packed with decidual leukocytes. The decidual stromal cells (DSCs) failed to differentiate into the large secretory DSC in these cases, remaining small and cuboidal and expressing lower levels of the nuclear progesterone receptor isoform B, and DSC markers Insulin Growth Factor Binding protein-1 (IGFBP-1) and CD10 as compared to controls. This study shows that defective progesterone mediated decidualization and a hostile maternal immune response against the invading endovascular EVT contribute to the failure of uterovascular remodeling in IUGR pregnancies.

## Introduction

Healthy pregnancies display uterine spiral arteries where the endothelial layer has been replaced with placentally-derived EVT and the muscular and elastic layers have been replaced with fibrinoid matrix. This results in their transformation from narrow, vasoactive arteries to flaccid, maximally dilated vessels unresponsive to vasomotor stimuli, and is necessary in order to increase maternal blood flow to the intervillous space for optimal fetal growth (1). Failed transformation of the spiral arteries in contrast is postulated as a contributing cause to several disorders of pregnancy including preeclampsia (PE), intrauterine growth restriction (IUGR), spontaneous preterm labor, preterm premature rupture of the membranes, and miscarriage (2–6). These cases are characterized by reduced utero-placental blood flow, documented by the observation of persistent high resistance waveforms using color/pulsed Doppler ultrasound of the proximal uterine spiral arteries (6–8). Such women do not exhibit any 2nd trimester fall in blood pressure (9) and placental bed biopsies (PBBx) from these subjects at delivery show failure of EVT invasion into the myometrial portions of spiral arteries despite adequate decidual interstitial EVT invasion (10–12). Histologic findings in these pathological PBBx represent varying combinations of the following features—collectively termed as decidual vasculopathy—on hematoxylin eosin stained sections; persistent muscularization of the spiral artery segments, a lack of endovascular trophoblast; apoptosis of endovascular EVT; a maternal leukocyte infiltrate, capable of inducing trophoblast apoptosis; atherosis and further narrowing of the vessel lumens or thrombosis in the spiral artery(2, 3, 5, 13–15). To date most studies have focused on the uteroplacental pathology of PE pregnancies while less is known about the uteroplacental pathology of IUGR. It is also unknown if this failure of uterovascular remodeling is primarily due to an EVT defect or in fact to a defect of decidualization. Recently, Brosens et al. have proposed that defective differentiation of the secretory endometrium to the decidua in the earliest stages of pregnancy may be a significant contributor to the development of the Great Obstetrical Conditions (16, 17).

Human decidualization occurs independently of pregnancy and is initiated following ovulation every menstrual cycle. In the mid-late secretory phase, the actions of progesterone, estradiol, and cAMP coordinate to promote the differentiation of the estrogen-primed endometrium into a receptive tissue prepared for blastocyst implantation in the event of fertilization. This process is referred to as decidualization and requires the coordination of a number of key events including; the differentiation of fibroblast-like endometrial stromal cells into large, rounded, secretory decidual stromal cells (DSC), the growth and elongation of the uterine spiral arteries, and the recruitment and specialization of a unique angiogenic decidual immune population (18, 19). The decidual leukocytes rapidly increase in number during the late secretory period to the effect that they comprise up to 40% of all decidual cells in the first trimester of pregnancy (20, 21). They are recruited from the periphery by chemokines secreted by the differentiated DSC, or proliferate within the decidua as it differentiates (22–24). The leukocyte populations include uterine natural killer cells (uNK, 50–70%), macrophage (20%), T-cells (10%), and myeloid dendritic cells (2%) and play a critical role in the development and transformation of the uterine spiral arteries (25–28).

Following fertilization and blastocyst attachment utero vascular remodeling is initiated early in gestation in both decidual and myometrial junctional zone segments of the spiral arteries. These changes occur prior to the presence of trophoblast in the tissue and are associated with disorganization of the perivascular smooth muscle cells (VSMC) layers, and coincide with the presence of large numbers of maternal leukocytes within the decidua (29–31). In decidual vessels undergoing active transformation numerous perivascular uNK and macrophages are found in close association with and infiltrating the disrupted VSMC layers both *in vivo* and *in vitro* (32–34). We have recently shown that these leukocytes secrete matrix metalloproteinases 2,−7,−9,−11,−16, and−19 which they utilize to disrupt ECM of the vascular wall (31, 35, 36). This leads to separation and disorganization of the VSMC layers and ultimately dedifferentiation and death of the VSMC (34, 37, 38).Vessels newly dilated by trophoblast-independent remodeling are further transformed and stabilized through trophoblast-dependent vascular remodeling, through which maximal dilation of the spiral arteries is finally achieved (39). We have suggested that the influx of decidual leukocytes into the vessel wall may also provide a chemokine stimulus to draw endovascular EVT up the vascular lumen to mediate the last stabilization of the transformed artery (19, 40). It has been shown that uNK secrete CCL8, CXCL-10, and CCL5 to promote EVT invasion via CXCR1 and CXCR3 receptors (40). Interestingly numbers of uNK are reduced in the decidua of term IUGR pregnancies (41), suggesting that altered uNK-EVT interactions may contribute to the failure of endovascular invasion associated with uteroplacental pathology.

The importance of leukocytes in the uterine vascular remodeling of the first trimester is well-established, yet new functions mediated by specific leukocyte populations and interactions between different decidual leukocyte populations and EVT are still being discovered (42). However, less is known about the second trimester decidual leukocyte populations although uterovascular transformation continues well into the 2nd trimester (43). In general studies support the development of a Th2 dominant tolerogenic immune environment in the second trimester under the control of rising levels of placental progesterone (44). Mechanisms employed by the various cell populations, including the DSC, act primarily to either reduce dNK cytotoxicity, or prevent activation of T-cell mediate immune responses either directly or indirectly by altering the phenotypes of antigen-presenting cells (macrophage and dendritic cells) (45, 46). We have previously shown that in the second trimester decidua macrophage differentiate to an alternate M2c proangiogenic tissue remodeling phenotype, T cells increase and are dominated by CD4 T helper cells and T-reg, and dendritic cells are maintained in an immature phenotype (27).

In this study we investigated the hypothesis that decidualization would be compromised in the 2nd trimester of pregnancies from women displaying high uterine artery pulsatile index (PI) and that this would be identified by both disturbances in the development, recruitment, and adaptation of the decidual leukocyte populations and decidualization of the DSC. We focused our investigation on 2nd trimester pregnancies carrying small for gestational age (SGA) fetuses in an aim to inform our observations in the 3rd trimester placental bed biopsies from IUGR pregnancies and thus contribute to the understanding of the development of uterovascular pathology in these cases.

## Methods

### Tissue Collection

This study was carried out in accordance with the recommendations of Mount Sinai Hospital Research Ethics Board, Sinai Health System. The protocol was approved by the Mount Sinai Hospital Research Ethics Board, Sinai Health System REB# 02-0061A and 12-0007E]. All subjects gave written informed consent in accordance with the Declaration of Helsinki. All research using human tissues was performed in a class II certified laboratory by qualified staff trained in biological and chemical safety protocols, and in accordance with Health Canada guidelines and regulations.

Placental bed biopsies were collected from healthy women undergoing elective caesarian section at term (*n* = 15) and from women with high uterine artery Doppler pulsatile index (PI > 1.5) undergoing caesarian section and carrying a IUGR fetus <10% expected fetal weight (*n* = 8). Five women with preeclampsia, a high uterine artery PI (>1.5) and carrying an IUGR fetus were also recruited for comparison. PI is calculated from measurement of (systolic velocity-diastolic velocity)/mean velocity of the uterine or umbilical artery. Biopsies were collected immediately following delivery of the fetus and placenta under direct visualization via the introduction of the punch biopsy forceps through the uterine incision. Three biopsies were collected; two from the placental site and one from the opposing wall of the uterus. Patient demographic data is presented in Table 1; placental pathology is presented in Table 2.

**Table 1:** Patient demographics of the placental bed biopsy study.

**Diagnosis**	**Control (*n* = 15)**	**IUGR (*n* = 8)**	**IUGR and PE (*n* = 5)**
No. of primigravid	2	1	0
Race (% white)	60%	50%	20%^*^
Umbilical artery Doppler	Normal, 15	AEDV, 3 REDV, 3 Increased PI, 2	AEDV, 4 Increased PI, 1
Uterine artery Doppler	Normal, 15	Increased PI, 7 Abnormal, 5 Not recorded, 1	Increased PI, 5 Abnormal, 1
No. of vessels examined	Decidual (44) Myometrial (56)	Decidual (21) Myometrial (19)	Decidual (12) Myometrial (13)
Maternal age, (year)	36 (4.3)	37.4 (4.7)	31.6 (5.1)
Systolic BP,(mm Hg)	104.07 (31.05)	120.0 (10.43)	148.80 (35.46)^*^
Diastolic BP, (mm Hg)	68.71 (11.86)	78.38 (8.42)	91.60 (11.08)^*^
Gestational age, week	>37, 15	<34, 6^*^ >37, 2	< 34, 4^*^ >37, 1
Birth weight, kg	3.3 (0.52)	1.30 (0.43)^*^	1.17 (0.36)^*^
Birth weight percentile	>50%, 15	5th−10th, 6 <5th, 2	5th−10th, 1 <5th, 4
Proteinuria	Abnormal 1, Not recorded, 14	Abnormal, 1 Not recorded, 8	Abnormal, 4 Not recorded, 1

A/REDV, Absent/reduced endovascular dopplar velocity. BP, blood pressure

* *P < 0.05 compared to healthy term controls*.

**Table 2:** Placental histopathology in cases and controls.

**Placental pathology**	**Normal (*n* = 15)**	**IUGR (*n* = 8)**
Small placenta <25%	0	5
Distal villous hypoplasia	0	2
Fetal thrombotic vasculopathy	1	3
Advanced villous maturity	0	3
Decidual vasculopathy	0	2
Multifocal infarction	0	3
>1 significant lesion	0	2
Villitis of unknown etiology	0	2
Umbilical cord abnormalities	1	4

Decidual tissue was obtained from healthy women undergoing elective termination for medical reasons between 14 and 19 weeks of gestation at the Second Trimester Interruption of Pregnancy (STIPS) clinic. Second trimester tissues were obtained via dilation and evacuation 24 h after insertion of a cervical laminaria. Uterine artery Doppler assessment was performed prior to the termination procedure. Of the second trimester cases collected, 6 had normal uterine artery Doppler and 6 had elevated uterine artery Doppler (PI > 1.6). Fetal size was also measured by ultra-sound (Table 3). The increase in the PI cut-off in this group is to account for the higher uterine artery PI normally observed at this gestational age (47).

**Table 3:** Patient demographics of the 2nd trimester decidua study.

**Diagnosis**	**Control (*n* = 6)**	**High Ut A PI (*n* = 6)**
Gestational age, week	14–19	17–19
Uterine artery Doppler	Normal, 6	Increased PI, 6 Abnormal, 5
No. of vessels examined	Decidual (22)	Decidual (34)
Maternal age, y	33 (2.3)	35.4 (3.1)
Systolic BP, mm Hg	119.23 (12.9)	120.0 (5.52)
Diastolic BP, mm Hg	76.71 (7.83)	80.12 (5.47)
Small for gestational age	0	4
**FETAL ABNORMALITIES:**		
Chromosomal	2	2
Genetic	2	1
Hydrops	1	0
Limb abnormalities	1	1
No heart beat	1	1
Severe IUGR	0	1

### Immunohistochemistry and Image Analysis

Serial 5 μm sections of each biopsy were immunostained using an avidin-biotin peroxidase technique as previously described (32). Antigen retrieval was performed by either heat mediated 10 mM sodium citrate (pH 6) solution or 1 mM EDTA (pH9) as determined by optimization studies and is indicated in Table 4. Blocking was performed for 1 h using Dako Protein Serum-Free Blocking Solution (Dako). Slides were then incubated with primary antibodies to identify EVT (CK7 and HLAG), myometrial and VSMC (Smooth muscle actin), endothelial cells (CD31), and decidual leukocytes (CD45) diluted in PBS or negative IgG controls (specific to the species in which primary antibodies were raised) and incubated overnight at 4°C. Further leukocyte subsets were investigated using antibodies against: CD56 (uNK), CD68 (macrophage), CD3, CD4, and CD8 (T Cells) and CD209 and CD83 (immature and mature dendritic cells). Antibodies are detailed in Table 4. Following incubation with species appropriate secondary biotinylated antibodies slides were washed with PBS, developed using the Liquid DAB+ (3,3-diaminobenzidine) Chromogen System (Dako), and counterstained with diluted Gill's No. 1 Hematoxylin (Sigma-Aldrich, Oakville, ON). True PBBx were confirmed by the presence of myometrium, decidua, one or more uterine spiral arteries, and positive staining for HLA-G or CK7 (indicating trophoblast presence). For negative controls, the specific primary antibody was replaced by normal mouse or rabbit IgG at the same antibody concentration used to stain test samples. No staining of the negative controls was observed. Terminal deoxynucleotidyl transferase dUTP nick end labeling (TUNEL) staining was performed using the in situ cell death detection kit-POD and according to the manufacturer's instructions (R and D systems, Oakville, Canada).

**Table 4:** Flow cytometry and immunohistochemistry antibody information.

**Antibody**	**Clone**	**Company (cat no)**	**Dilution/Ag Retrieval**	**Specificity**
CD45 APC-Cy7	2D1	BD 557833	1:33	Common Leukocyte Antigen
CD3 FITC	HIT3a	BD 555339	1:25	T Lymphocytes
CD56 PE-Cy7	B159	BD 557747	1:33	Uterine Natural Killer Cells (NCAM)
CD68 PE	Y1-82A	BD 555743	1:6.6	Mac-1 Integrin (Activation Marker)
CD209 PerCP-Cy5.5	DCN-46	BD 558263	1:10	Immature Dendritic Cells (DC)
CD205 PE	MG38	BD 558069	1:25	Intermediate/Antigen Uptake Receptor DC
CD83 APC	HB15e	BD 551073	1:5	Mature Antigen Presentation DC
CK-7	OV-TL 12/30	Dako M7018	1:200 1 mM EDTA pH9. Heat	Epithelial Cells
HLA-G	4H84	Exbio 11-499-C	1:300 10 mM Na Citrate pH6. Heat	Extravillous Trophoblast
SMA	1A4	Dako M0851	1:100 10 mM Na Citrate pH6. Heat	Smooth Muscle Cells
CD31	JC70A	Dako M0823	1:50 10 mM Na Citrate pH6. Heat	Endothelial Cells
CD45	2B11+PD7/26	Dako M0701	1:100 10 mM Na Citrate pH6. Heat	Common Leukocyte Antigen
CD56/NCAM	123C3	Dako M7204	1:50 1 mM EDTA pH9. Heat	Uterine Natural Killer Cells
CD68	PG-M1	Dako M0876	1:200 10 mM Na Citrate pH6. Heat	Macrophage
CD3	F7.2.38	Dako M7254	1:50 1 mM EDTA pH9.Heat	T cells
CD4	EPR6855	Abcam Ab133616	1:200 10 mM Na Citrate pH6. Heat	T helper cells
CD8	EP1150Y	Abcam Ab93278	1:200 10 mM Na Citrate pH6. Heat	Cytotoxic T cells
CD209	120612	R and D systems MAB1621	1:200 1 mM EDTA pH9. Heat	Immature DC
CD83	HB15e	E Bioscience 14-0839-82	1:50 10 mM Na Citrate pH6. Heat	Mature Antigen Presentation DC
D240/podoplanin	730-01	Covance SIG-3730	1:50 1 mM EDTA pH9. Heat	Lymphatic endothelium
IGFBP-1	H-120	SC Biotech sc-13097	1:200 10 mM Na Citrate pH6 Heat	IGF binding protein 1
CD10	97C5	SC Biotech sc-19993	1:50 1 mM EDTA pH9. Heat	Common acute lymphocytic leukemia antigen
Mouse IgG	DAK-501	Dako X0931	1:300	Mouse Fc
Rabbit IgG	Rabbit Ig fraction	Dako X0903	1:300	Rabbit Fc

### Analysis of Structural Alterations in the Placental Bed

Structural alternations in the placental bed biopsies were scored based on previous publications (14, 48). Sections were scored by 2 observers (C.D. and S.K.) blinded to the sections identity and showed good agreement between observers. Decidual and myometrial vessels were considered separately. Disruption of the vessel wall was graded into 3 groups (preserved, disorganized (disorganization and some loss of the media) and absent/grossly disorganized (little or no media remains). Interstitial EVT (inEVT) found > 50 μm from the vessel wall were graded as absent (0), low density (1) or high density (2). Intramural EVT (mEVT) and endovascular EVT (enEVT) were scored as present or absent if within the vessel media, or where it once was, or lumen, respectively.

### Image Analysis and Quantification

Quantification of leukocyte subtypes and decidual markers in the PBBx samples controls (*n* = 15 basalis, *n* = 15 paretalis) and IUGR (*n* = 8) and IUGR with maternal PE (*n* = 5) or second trimester human decidual samples from pregnancies with high uterine artery PI (*n* = 6) or normal uterine artery PI (*n* = 6) was performed using an Olympus BX61 microscope equipped with an Olympus DP72 camera, and the newCAST software (Visopharm). Firstly 2 separate masks were established covering the decidual and myometrial areas of the PBBx section. Counts were performed using a standard protocol that assigned random counting frames covering 5% of each total masked tissue area. Brown, positively-staining cells and blue, negatively-staining cells (haematoxylin-stained) were counted at 10X magnification. A positively stained ratio was generated by dividing the total numbers of brown, positively-staining cells by the total number of cells counted in the tissue area).

### Isolation of Decidual Leukocytes

Decidual tissue was processed as described in Kwan et al. (27). Briefly, decidua was washed and minced finely and flushed with HBSS^+/+^ 2 to 3 times to ensure maximal release of leukocytes. Decidual cells were collected via filtration through 100 and 70 μm sieves and centrifugation (700 g for 10 min at 4°C). Isolated cells were suspended in RPMI-1640 + 10% FBS and fibroblasts eliminated through differential attachment to tissue culture plates (37°C for 20 min). Following incubation, remaining cells were passed through a 40 μm filter and incubated in erythrocyte lysis buffer EL (Qiagen, Toronto, ON, Canada) for 20 min at 4°C. The final cell suspension was incubated in serum-free protein block (Dako, Burlington, ON, Canada) for 1 h on ice and diluted to a final concentration of 10^6^cells/mL.

### Cell Labeling and FACS Analysis Gating Strategy

Isolated cells were incubated with fluorochrome-conjugated mouse monoclonal antibodies in 200 μl staining volume for 45 min at 4°C in the dark. Cells were washed in PBS and re-suspended in 200 μl stabilizing-fixative permeabilization buffer (BD Biosciences, Mississauga, ON, Canada) to prevent dissociation of tandem dyes and analyzed using a FACSAria flow cytometer (BD Biosciences, Mississauga, ON, Canada) and FlowJo software (Tree Star, Ashland, OR, USA). Cell debris and aggregates were excluded by gating on forward vs. side scatter, then by height vs. width of forward and side scatter plots prior to population discrimination. Non-leukocyte events were excluded from analysis using threshold gates set on CD45 fluorescence. Positive leukocyte subpopulations were identified upon comparison of fully-stained samples to fluorescence minus one (FMO) and isotype controls. Dendritic cell populations were assessed in more detail within the high side scatter gate within the CD45 positive leukocyte population. Antibodies used are listed in Table 4.

### Statistical Analysis

Outliers were identified using Grubb's test and excluded from subsequent analysis. Prism 5 (GraphPad, La Jolla, CA, USA) was used to perform ANOVA or unpaired Student's *t*-tests assessing changes in means ± standard deviation of the mean (SD) of patient demographics compared to control term pregnancies and leukocyte populations between second trimester normal and high uterine artery PI cases. Data was tested for normality prior to ANOVA analysis Leukocyte populations were quantified as a percentage of total CD45^+^ leukocytes except where specified; *p* < 0.05 or greater was considered statistically significant.

## Results

### Failure of Uterine Vascular Transformation Is Associated With Apoptotic EVT and Altered Maternal Immune Status in IUGR Placental Bed Biopsies

In comparison to healthy term pregnancies in the control group (Normal, *n* = 15) the idiopathic IUGR patient group had; significantly higher uterine artery pulsatile indices (PI), absent (AEDV) or reversed (REDV) umbilical end-diastolic velocity in their umbilical artery, babies born at <10th %tile of weight for gestational age, and placental pathology indicative of IUGR (*n* = 8). A further 5 cases of severe IUGR were collected from women with preeclampsia defined according to the ACOG criteria, showing elevated maternal blood pressure, proteinuria and indicators of end organ damage (Tables 1, 2).

**Figure 1:**
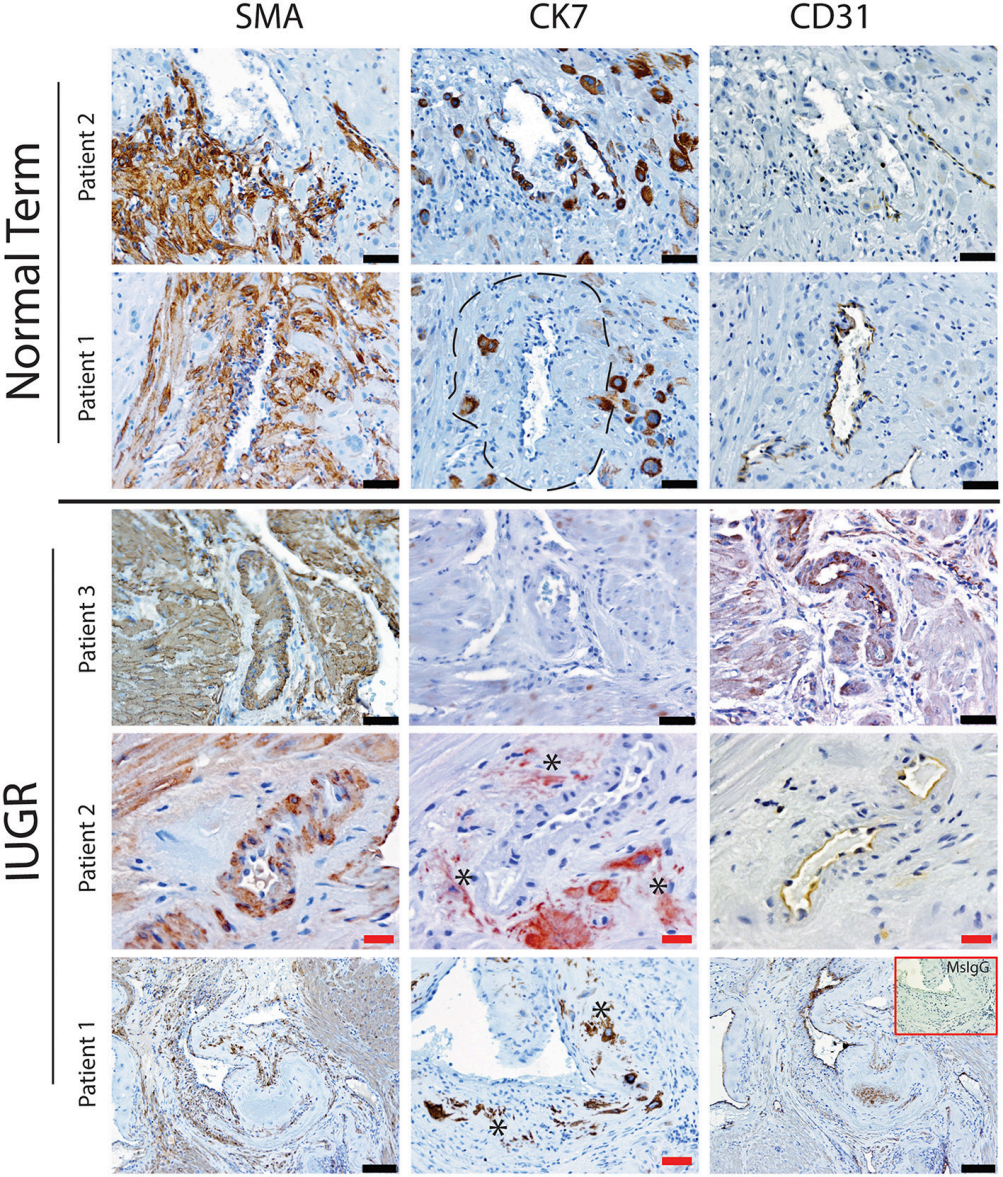
Failed uterovascular transformation in IUGR is associated with persistance of vascular media and failure of interstitial and endovascular invasion. Immunohistochemical staining was performed to determine the degree of uterine artery transformation in PBBx biopsies using antibodies against smooth muscle actin (SMA, Column 1) cytokeratin 7 (CK7, Column 2) and PECAM (CD31, Column 3). Representative serial sections of control (*n* = 2/15) and IUGR PBBx (*n* = 3/8) uterine spiral arteries at the decidual myometrial junction are shown. Asterisks denote the presence of fragmented likely apopototic mural EVT in the IUGR cases. Fibrin deposition in the fully transformed artery is shown in patient 1 Normal marked by the hatched line. Negative control mouse IgG1 is shown in the inset lower right. Scale bars: black = 50 μM, 100x magnification; red = 20 μM, 200x.magnification.

**Figure 2:**
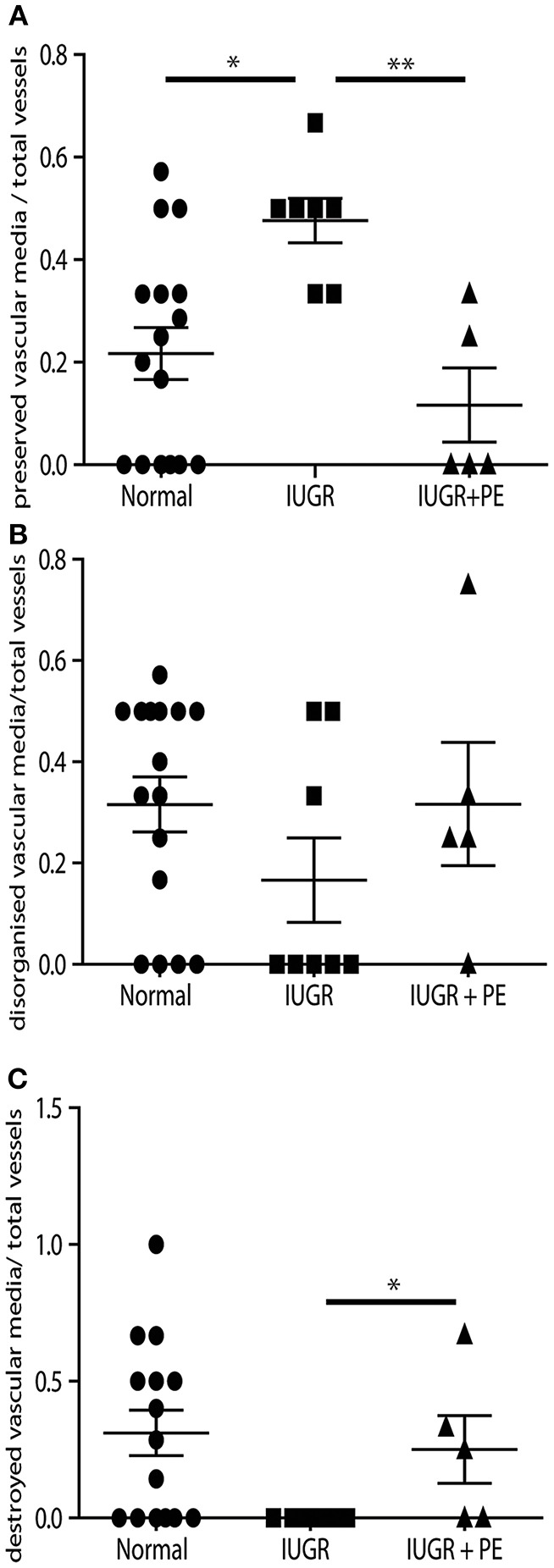
IUGR PBBx display high numbers of preserved uterine spiral arteries. Graphs show quantitation of the degree of remodeling of all myometrial vessels control (circle), IUGR (square) and IUGR with preeclampsia (triangle) groups. Preserved vessels were scored when intact multi layered vascular smooth muscle media was present **(A)**. Disorganized vessels were scored when the vascular media was separated and smooth muscle cells were migrating away from the outer layers of the media **(B)**. Destroyed vessels were scored when all media had been lost and replaced with fibrin **(C)**. *p < 0.05, **p < 0.01.

Uterine spiral arteries in the myometrial decidual junction of PBBx from healthy term pregnancies displayed significant disorganization and complete disruption of their vascular smooth muscle (VSMC) media and loss of the endothelium, associated with the presence of both enEVT and inEVT (Figure 1, Patient 1). Full transformation was associated with mEVT and fibrin deposition in the arterial media (marked area, Patient 2). In contrast, and as expected, PBBx from IUGR pregnancies showed a higher number of preserved uterine arteries with thick organized vascular smooth muscle media and intact endothelium (Figure 1, Patients 1–3). Quantitation of all myometrial vessels across the patient subgroups showed that showed that the IUGR biopsies had significantly higher numbers of preserved arteries and a significantly lower number of destroyed vessels compared to controls (*p* < 0.05, Figure 2). This was associated with a lack of both inEVT and enEVT invasion into the myometrium (Supplemental Figure 1A and Tables 5, 6). Placental bed biopsies from IUGR and preeclamptic pregnancies showed a similar lack of enEVT invasion but surprisingly inEVT was unaffected and vascular transformation was not as compromised (Figure 2, Tables 5–7, and Supplemental Figure 1A). Scoring of decidual vessels and trophoblast invasion showed a small increase in preserved vessels and a low number of interstitial EVT in the IUGR samples as compared to healthy controls (Table 5 and Supplemental Figure 1B).

**Table 5:** Interstitial EVT are reduced in the IUGR myometrium and decidua.

**Interstitial EVT**	**Myometrium**			**Decidua**	
**% of vessels**	**Absent**	**Low**	**High**	**Low**	**High**
Normal	0.00	57.14	42.86	54.55	40.91
IUGR	**89.47**	10.53	0.00	**66.67**	33.33
IUGR and PE	0.00	30.77	69.23	33.33	**66.67**

*Data is presented as a percentage of all vessels in each PBBx section. Bold text indicates a significant difference from non-bold text, assessed by unpaired T-test with Welch's correction, P < 0.05*.

**Table 6:** Endovascular EVT are absent in IUGR and IUGR and PE myometrium.

**Endovascular EVT**	**Myometrium**		**Decidua**	
**% of vessels**	**Present**	**Absent**	**Present**	**Absent**
Normal	32.14	67.86	61.36	38.64
IUGR	15.79	**84.21**	57.14	42.86
IUGR and PE	7.69	**92.31**	50.00	50.00

*Data is presented as a percentage of all vessels in each PBBx section. Bold text indicates a significant difference from non-bold text, assessed by unpaired T-test with Welch's correction, P < 0.05*.

**Table 7:** Intramural EVT are reduced in the IUGR decidua and myometrium.

**Intramural EVT**	**Myometrium**		**Decidua**	
**% of vessels**	**Present**	**Absent**	**Present**	**Absent**
Normal	37.50	**62.50**	**75.00**	25.00
IUGR	31.58	**68.42**	52.38	47.62
IUGR and PE	15.38	**84.62**	**83.33**	16.67

*Data is presented as a percentage of all vessels in each PBBx section. Bold text indicates a significant difference from non-bold text, assessed by unpaired T-test with Welch's correction, P < 0.05*.

**Figure 3:**
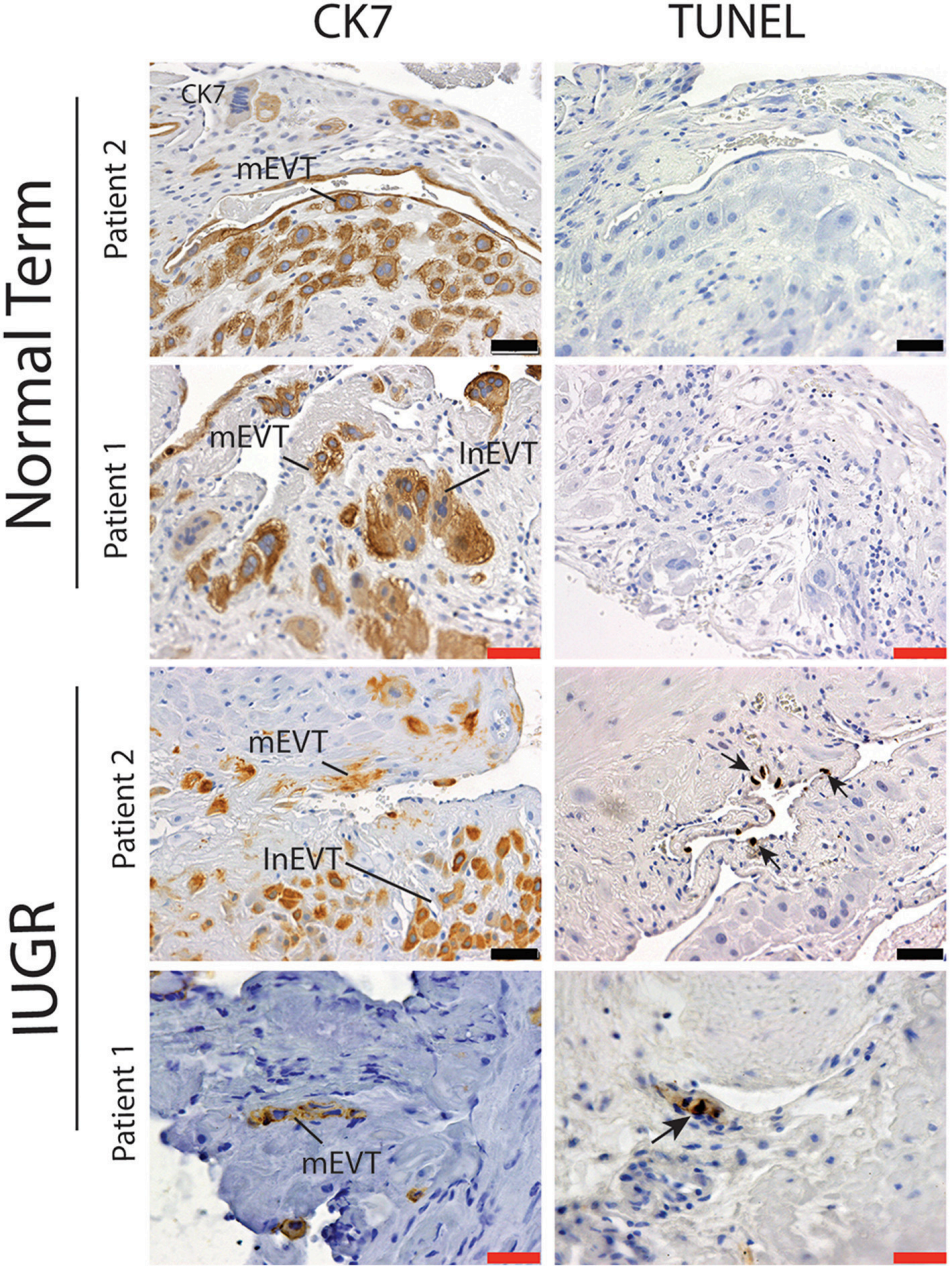
Failed uterovascular transformation in IUGR is associated with the presence of apoptotic mural EVT. Immunohistochemical staining for cytokeratin 7 (CK7, Column 1) and Terminal deoxynucleotidyl transferase dUTP nick end labeling (TUNEL, Column 2) was perfomed to confirm that the fragmented mural EVT that we observed in the IUGR cases were apoptotic. Representative serial sections of control (*n* = 2/15) and IUGR PBBx (*n* = 2/8) uterine spiral arteries at the decidual myometrial junction are shown. Arrows indicate the TUNEL positively nuclei of the mural EVT (mEVT) showing cytokeratin loss in the matching serial sections in the IUGR samples. No TUNEL staining was seen in the interstitial EVT (inEVT) of the IUGR cases or any of the EVT populations of the control term samples. Scale bars: black = 50 μM, 100x magnification; red = 20 μM, 200x.magnification.

Interestingly numbers of uterine arteries with an absence of mEVT were equal between controls and pure IUGR samples (Table 7). However, in the IUGR cases these mural EVT often displayed signs of fragmentation, nuclear condensation, blebbing, and loss of cytokeratin immunoreactivity, indicative of apoptosis (Figure 1, IUGR patient 2 and 3, asterisks). TUNEL staining confirmed that these mural EVT were indeed apoptotic (arrows) showing positive staining of condensed nuclei in the IUGR cases, however the inEVT in the decidua were unaffected. Controls did not show any TUNEL reactivity in any of the EVT subtypes (Figure 3).

**Figure 4:**
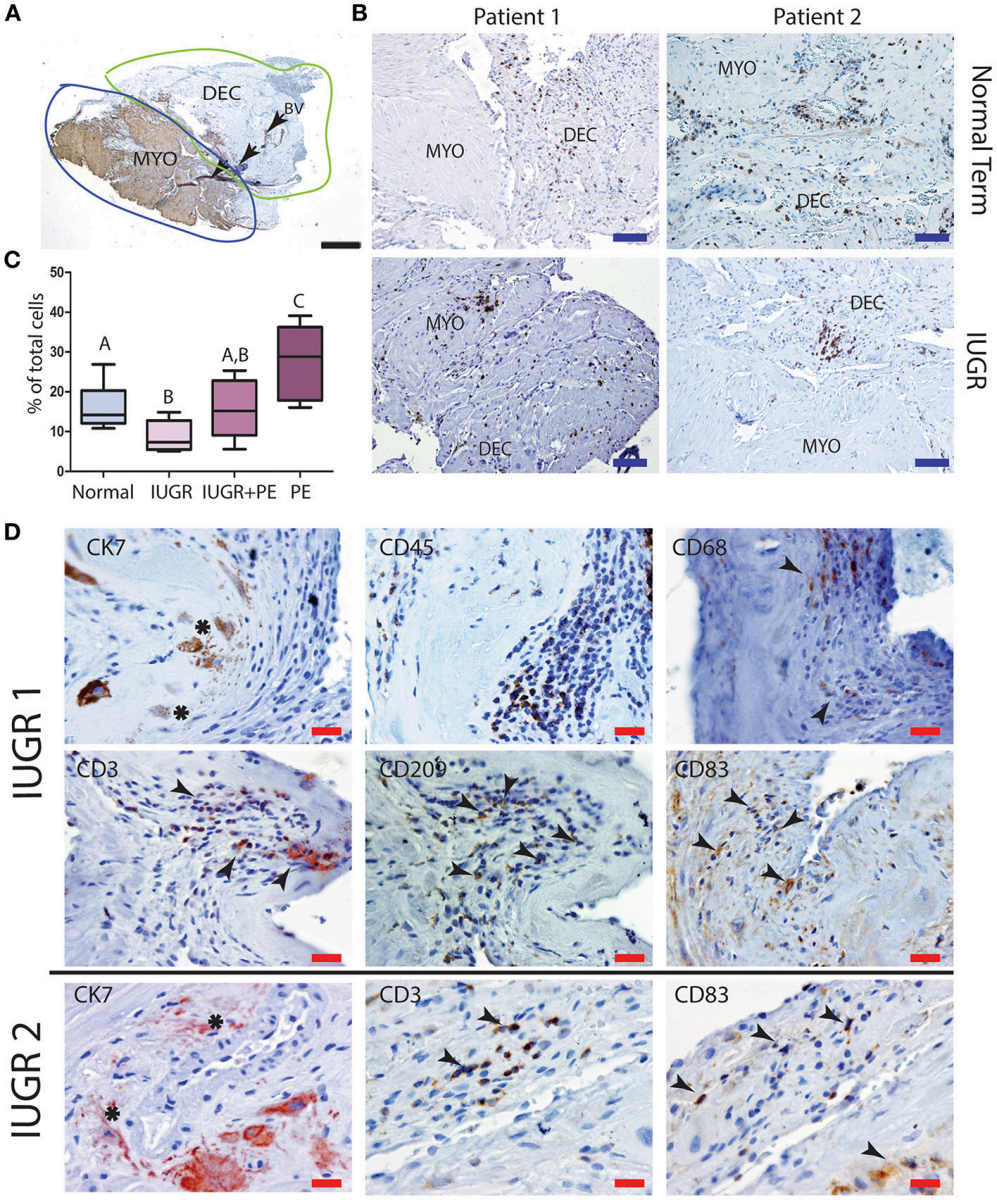
Low decidual leukocyte number and increased mature dendritic cells in association with apoptotic EVT in IUGR. Visiopharm Newcast image analysis system was used to define decidual and myometrial regions based on smooth muscle actin staining of the myometrium **(A)**. Representative stained decidual and myometrial sections from normal term PBBx upper panel and IUGR PBBx lower panel **(B)**. Numbers of brown stained CD45+ leukocytes and blue hematoxylin stained decidual stromal cells or myometrial muscle cells were counted in corresponding serial sections across 5% of the respective region using Visiopharm Newcast image analysis system and random meander sampling **(C)**: Normal *n* = 15, IUGR *n* = 8, IUGR and PE *n* = 5, PE *n* = 8) (^A,B^*p* < 0.01, ^A−C^*p* < 0.0001, ^B,C^*p* < 0.001). Representative serial sections of IUGR PBBx show that the apoptotic mural EVT (CK7, asterisks) that were observed in these biopsies were associated with leukocyte clusters (CD45) containing CD68+ macrophage and elevated number of CD3 T cells and CD83+ mature dendritic cells (**D**, arrows). Scale bars: black = 500 μM, 20x magnification, blue = 250 μM, 40x magnification, red = 20 μM, 200x.magnification.

Further investigation of the maternal leukocyte populations in the PBBx was initially undertaken by immunostaining and image analysis to count the numbers of CD45^+^ leukocytes present in the decidua vs. the myometrium (Figure 4A). Predominantly leukocytes were found in the decidua, while the few leukocytes observed in the myometrium were usually associated with the vascular plexus of the uterine vasculature (Figure 4B). When we compared the biopsies from the placental site (decidua basalis) with the non-placental site (decidua paretalis in the control samples we found there was no difference in the number of CD45+ leukocytes (data not shown). In contrast the numbers of CD45+ leukocytes were shown to be significantly lower in the IUGR decidua basalis as compared to control (*p* < 0.001) (Figure 4C). There was no significant difference between IUGR and IUGR complicated by preeclampsia. However, decidua from severe early onset preeclampsia with a normal weight baby (for gestational age) showed a significantly higher number of leukocytes in comparison to all other groups (*p* < 0.0001). In serial sections CD45^+^ leukocytes were more commonly observed in dense clusters in the IUGR placental bed biopsies, and in close association with the apoptotic mural EVT (Figure 4D). Identification of the immune cell subtypes in these clusters showed the presence of immunoreactive CD68^+^ macrophage, CD3^+^ T cells and both immature CD209^+^ and mature CD83^+^ dendritic cells (arrows) (Figure 4D, IUGR Patient 1 and 2 matches to Figure 1). In control placental bed biopsies leukocytes were not found in association with mural EVT in the fibrin matrix of transformed vessels and mature CD83 dendritic cells were very infrequent (Supplemental Figure 2).

### Failed Decidualization and Immune Dysregulation in the 2nd Trimester Is Associated With High Uterine Artery PI and Growth Retardation

To gain insight into the origin of the failed vascular transformation and potential immune dysregulation observed in the third trimester IUGR samples we collected decidua from women undergoing 2nd trimester termination for medical reasons. Prior to the surgical procedure uterine artery Doppler analysis was performed. Patients were grouped by uterine artery Doppler indices specific to the 2nd trimester into normal (*n* = 6), and high uterine artery PI (>1.6) with early end-diastolic notches (*n* = 6) which has been shown to be associated with an increased incidence of preeclampsia at this time point in gestation (49). Fetal abnormalities were chromosomal (T21, T18), genetic (Turners syndrome, Amniotic band syndrome, or 48XY deletion) or no fetal heartbeat. Interestingly when patient data was collated 4 of the 6 high uterine artery PI group had small for gestational age (SGA) fetuses (fetal size was assessed crown to rump length by ultrasound and was noted as expected size of 2 weeks earlier than actual gestational age). In this study we have designated this group as high uterine artery PI and small for gestational age (SGA). We do however acknowledge that we cannot exclude an effect of the fetal abnormality on the fetal size due to the small size of the patient groups; only one pregnancy was terminated at 18-weeks for pure severe IUGR. The control group had normal uterine artery indices and all fetuses were of the expected size for gestational age. Equal numbers and types of fetal abnormality were found in each group (Table 3).

**Figure 5:**
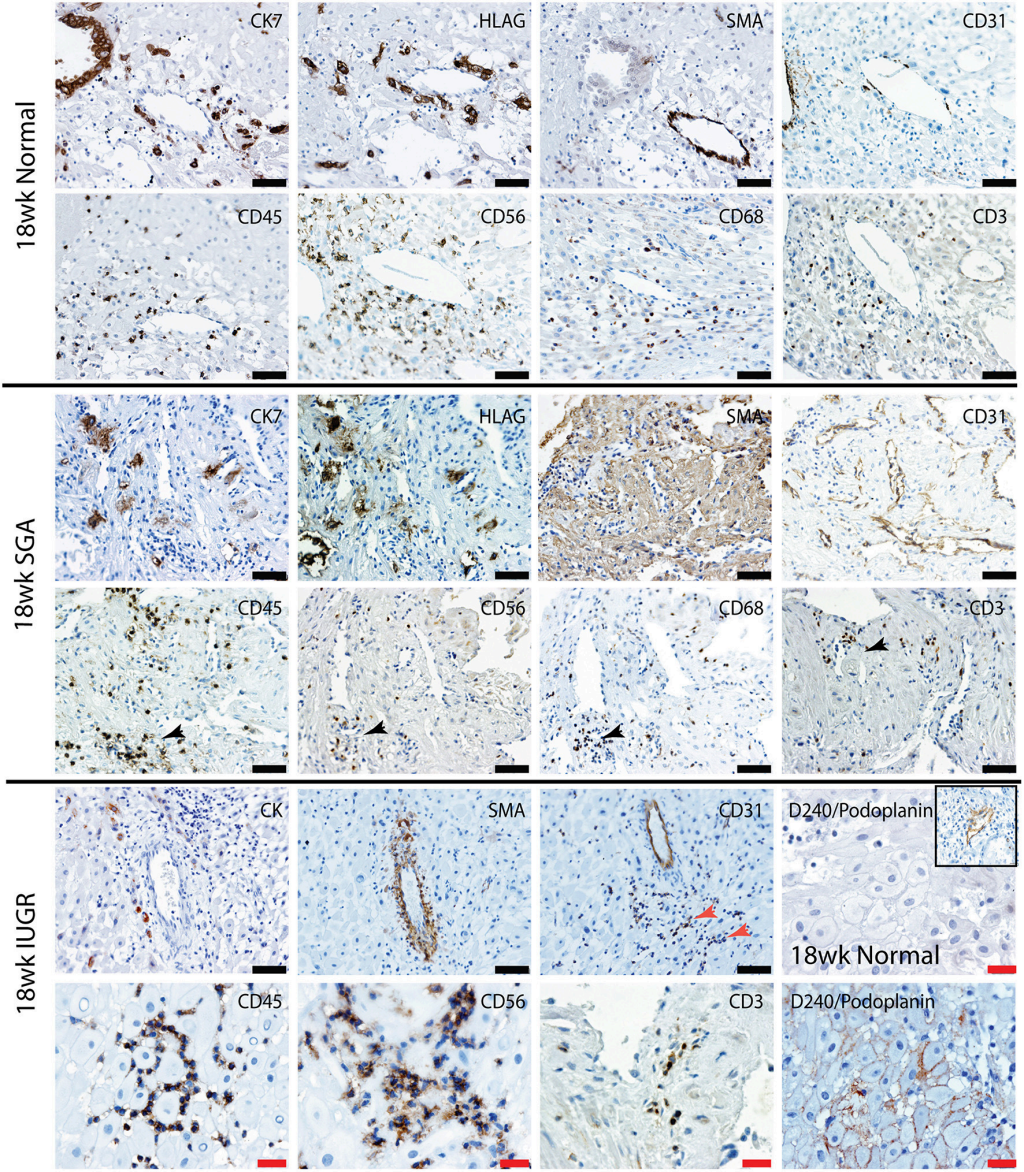
Lymphatic vessel density is increased, and leukocytes are abnormally distributed in the high uterine artery PI SGA decidua. Representative serial sections from normal 18 week trimester decidua (top panel), an 18 week high uterine artery PI SGA case (middle panel) and a 18 week high uterine artery case of IUGR (lower panel) stained with antibodies against CK7 (trophoblast) HLAG (EVT), SMA (VSMC), CD31 (endothelial), CD45 (leukocytes) CD56 (uNK) CD68 (macrophage and CD3 (T cells). Lymphatic vessels were identified by staining with D240/podoplanin (lower right 2 images control and IUGR). In the SGA high numbers of small CD31 vessels full of leukocytes were seen (black arrows) In the IUGR decidua this was more pronounced (red arrow), abnormal D240 podoplanin lymphatic endothelial expression was found surrounding the DSC (lower right panel) creating micro-lymphatic vessels that were packed with uNK (CD56) and T cells (CD3). In controls the DSC were negative and only the larger lymphatic vessels stained positive (inset). Scale bars: black = 50 μM, 100x magnification, red= 20μM, 200x magnification.

Uterovascular remodeling and 2nd trimester decidual leukocyte populations were assessed by immunolocalization, image analysis and multi-color flow cytometry studies. In the 2nd trimester control group interstitial trophoblast staining positively for both CK7 and the EVT marker HLA-G were observed in the walls of Stage III actively remodeling decidual arterioles that had disrupted VSMC and lost their endothelial cells (Figure 5, top panel 18 weeks normal). Fully transformed Stage IV vessels were also observed lined with enEVT. As we have previously shown in the first trimester (34), this active vascular transformation was associated with the presence of uNK and macrophage within the vessel wall (lower panel 18 weeks normal). Significant numbers of CD3^+^ T cells were also observed in association with the vessels.

In contrast the decidua from the high uterine artery PI SGA group was characterized by the presence of InEVT in association with the vasculature, but no enEVT were observed (Figure 5, lower 2 panels). In 2 SGA cases abnormal smooth muscle actin staining was seen in the DSCs and arterioles maintained their VSMC wall and intact endothelium. Most importantly, we consistently observed an increased number of small lymphatic vessels leukocytes marked by both CD31 and D240/podoplanin expression (black arrow, SGA and red arrow, IUGR). Moreover, abnormal D240/podoplanin expression lining the DSCs was also observed in the high uterine artery and SGA cases These micro-lymphatics between the DSC are densely packed with CD56^+^ uNK, CD68^+^ macrophage and CD3^+^ T cells (Figure 5, 18 weeks SGA and high magnification 18 weeks IUGR). Indeed, the majority of the decidual leukocytes appear to be restricted within the lymphatic vasculature in the high PI SGA cases and were not distributed in the decidual stroma, or associated with transforming vessels, as they are in normal pregnancies. In the IUGR case trophoblast were also observed within the lymphatic vasculature (CK, arrow).

**Figure 6:**
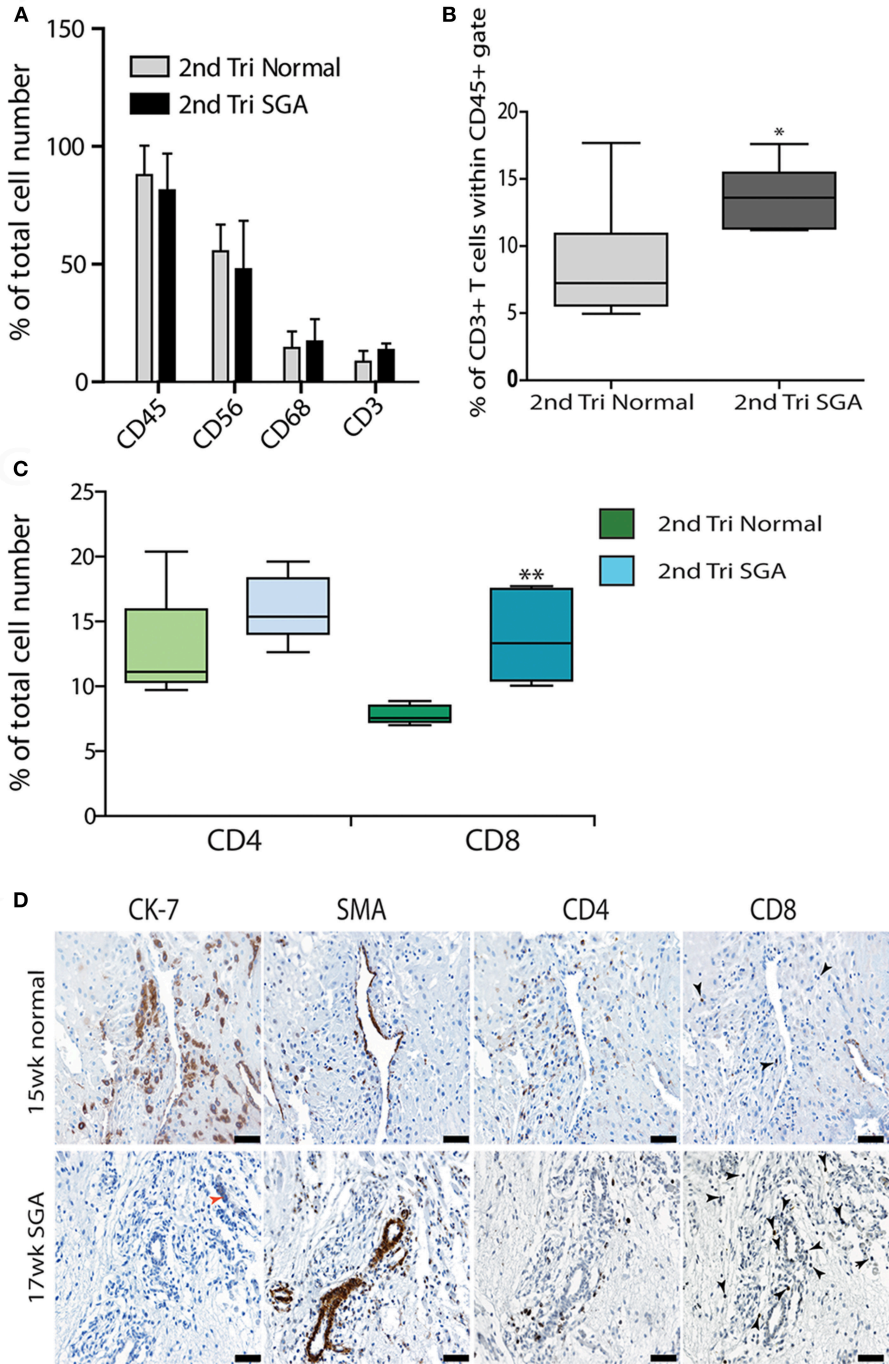
Elevated cytotoxic CD8^+^ T cells in 2nd trimester high Uterine artery PI and SGA decidua. Multicolored flow cytometry analysis of leukocyte subsets isolated from 2nd trimester normal or high uterine artery PI (>1.6) of decidua. Numbers of CD45^+^ leukocytes, CD56^++^ uNK, and CD68^+^ macrophage were no different between groups **(A)**. CD3+ T cells gated within the CD45^+^ population were doubled in the high uterine artery PI decidua **(B)**. Visiopharm Newcast image analysis system and random meander sampling was used to quantify numbers of CD4^+^ T cells and CD8^+^ T cells in 5% of the total tissue area of each decidual section. Percentages of the total cell number were calculated and are presented as box whisker plots in **(C)**. Representative serial sections from a 15-week normal sample (upper panel) and a 17-week high uterine artery PI SGA) stained with CK7, SMA CD4 and CD8 antibodies are shown in **(D)** (arrows identify CD8+ T cells). *p < 0.05, **p < 0.01, *n* = 6 in each group. Scale bars = 20 μM, 200x magnification.

Further investigation of the decidual leukocyte subsets by flow cytometry showed that numbers of total CD45^+^ leukocytes, CD56^+^ uNK, and CD68^+^ macrophage were not different between normal and high uterine artery PI SGA groups and conformed to expected ratios in the second trimester when gated within either total live cells or the CD45^+^ leukocyte population (CD56^+^ uNK at 50–60% and CD68^+^ macrophage at 10–20%, Figure 6A). In contrast, numbers of CD3^+^ T cells were doubled in the high uterine artery with SGA fetuses' groups as compared to the controls (13.57 vs. 7.23%, *p* < 0.01, Figure 6B) when gated within the CD45^+^ population. This significant increase was not seen in the total cell comparison, probably due to an increase in numbers of small DSCs in the high uterine artery PI SGA group (observed in the image analysis of DSCs described below). The T cell subpopulations were further investigated by immunostaining serial sections of decidua with anti-CD4 and antiCD8 antibodies and quantifying T cell number using image analysis. In comparison to controls the high uterine artery PI SGA group had significantly higher numbers of cytotoxic CD8^+^ T cells (*p* < 0.001, Figure 6C) and a significantly decreased CD4/CD8 ratio (1.61 ± 0.39 vs. 1.212 ± 0.35, *p* = 0.0028). Both CD4^+^ and CD8^+^ T cells were found in increased density in the SGA cases surrounding untransformed uterine spiral arteries (arrows, Figure 6D). In the controls CD4^+^ T cells were associated with the transforming vessels but CD8^+^ T cells were very small and infrequent Figure 6D, arrows).

**Figure 7:**
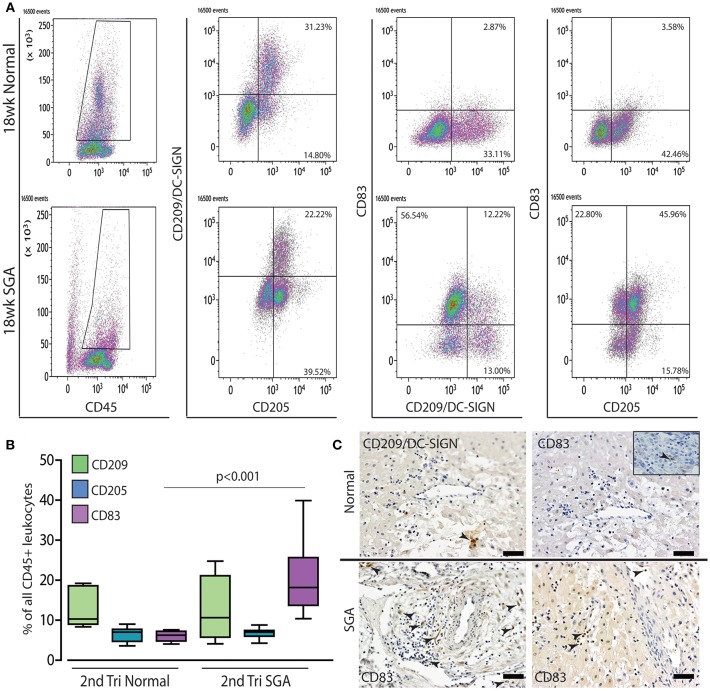
Mature dendritic cells are significantly increased in the 2nd trimester high uterine artery SGA decidua. Representative dot plots of multicolor flow analysis of decidual dendritic cells from an 18-week normal decidua and a decidua from an 18-week high uterine artery PI SGA case. Dendritic differentiation was assessed using three markers CD209/DC-SIGN to mark immature dendritic cells, CD205 to mark intermediate dendritic cells and CD85 to mark mature antigen presenting dendritic cells. Gates were set in the high granular region of the CD45+ gate **(A)**. Comparison across all samples showed that mature CD83+ dendritic cells are increased to 20% of all CD45^+^ leukocytes compared to 4%+- in the controls **(B)** (*p* < 0.001, *n* = 6 per group). Representative immunostained images from one control and 2 high uterine artery PI SGA cases, CD209: immature dendritic cells, CD83: mature antigen presenting dendritic cells (**C**, arrows indicate positively stained cells). Inset shows rare CD83^+^ dendritic cell in control decidua. Scale bar = 20 μM, 200x magnification.

The maturation status of decidual dendritic populations was assessed in more detail in the high granular CD45^+^ gate by flow cytometry using CD209/DC-SIGN (immature), CD205 (intermediate), and CD83 (mature antigen presenting) antibodies. A representative comparison of 18 weeks normal and high Uterine artery PI SGA cases is shown in Figure 7A. As can be seen, and as we have previously shown (27), dendritic cells in the normal 2nd trimester decidua are maintained in an immature state only expressing CD209 and CD205. In contrast the high uterine artery PI IUGR case shows the appearance of a significant CD205^+^ CD83^+^ population that is absent in the control (45.96 vs. 3.58%). Quantification within the CD45 gate across all cases in the respective groups showed that the increase in numbers of CD83^+^ mature dendritic cells was common to the high Uterine artery PI SGA group (Figure 7B, *p* < 0.001). These CD83^+^ dendritic cells were observed in leukocyte clusters in association with unremodeled vessels and in lymphatic vessels in the high uterine artery PI SGA cases (Figure 7C, SGA, arrows).

**Figure 8:**
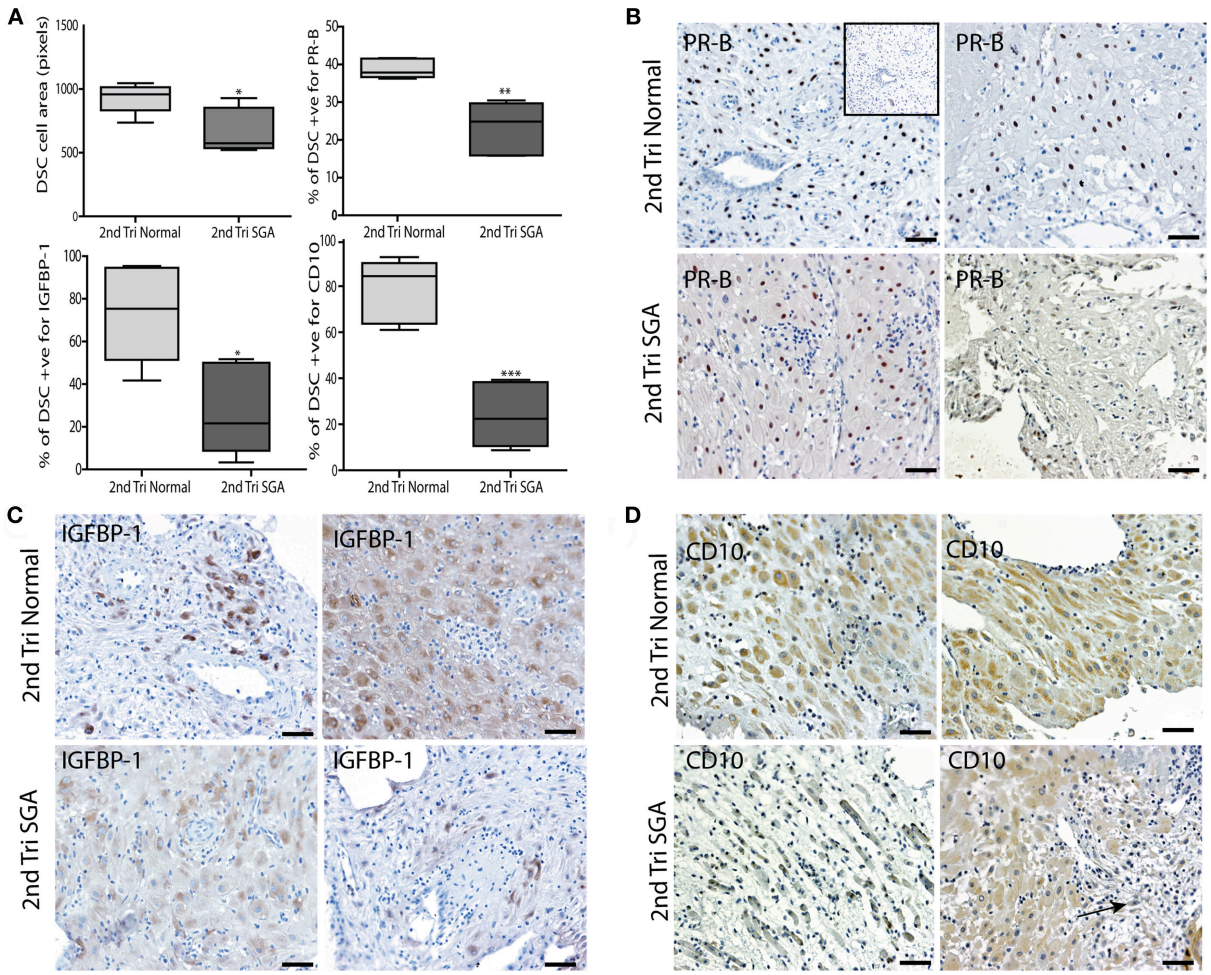
DSC differentiation fails in the 2nd trimester high uterine artery PI SGA decidua. **(A)** DSC cell area was measured using the random ellipsoid measurement tool from the Olympus Cellsens software. Immunohistochemical image analysis was performed on serial decidual sections stained with antibodies against the decidual markers PR-B, IGFBP-1, and CD10. Visiopharm Newcast image analysis system and random meander sampling was used to quantify numbers of positively stained DSC in 5% of the total tissue area of each decidual section. Percentages of the total cell number were calculated and are presented as box whisker plots in **(A)**. Representative photographs of serial sections from 2normal 2nd trimester decidua (top panels), and 2 high uterine artery PI SGA cases (lower panels) in each case are shown stained with PR-B **(B)**, IGFBP-1**(C)** and CD10 **(D)** antibodies (arrow indicates area of increased lymphatic vessel density). *p < 0.05, **p < 0.01, ***p < 0.0001, *n* = 6 in each group. Inset shows no staining with the Rabbit IgG negative control. Scale bar = 50 μM, 100x magnification.

Correlation of the above results supported our hypothesis that the initial failure of early decidualization may underlie the resulting uteroplacental pathology of severe IUGR. To investigate this final step in our hypothesis we investigated DSC differentiation status using 3 markers. Progesterone is known to play a key role in the decidualization of DSCs by binding to its nuclear receptor PR-B (50). IGFBP-1 is expressed by DSC as they differentiate to their secretory phenotype (51) and CD10 is expressed by mature DSC (52). To investigate if lower levels of these DSC markers correlated with the above changes in the high uterine artery PI decidua, we performed immunostaining and image analysis to count the numbers of PR-B positive nuclei in the DSC or quantify the intensity of staining in the IGFBP-1 and CD10 slides. DSC size was also quantified by ellipsoid area measurements of 3 random fields of each decidual section (Figure 8A). Control 2nd trimester decidua was characterized by large plump pale DSC with large pale nuclei, known to be indicative of a highly active cell. In contrast, the decidual cells of the decidua were physically much smaller, stained more intensely with hematoxylin and displayed small dark staining nuclei. Area measurements showed that control DSC were much larger than decidual cells from the high uterine artery PI SGA cases (930.8 ± 116.6 vs. 670.7 ± 175.9, *p* < 0.05). In the 2 SGA cases showing the decidua also displayed elongated rectangular cells arranged in bands separated by ECM that also expressed abnormal focal points of SMA (Figure 8D). Many gaps were also observed between the decidual cells in the high uterine artery PI decidua corresponding to the elevated number of lymphatic vessels reported above (Figure 8D, arrow). In the markers of decidualization analysis more DSC expressed strong nuclear PR-B staining in the control normal samples (38.84 ± 2.57% vs. 23.56 ± 6.66%, *p* < 0.0001) (Figure 8C). Interestingly IGFBP-1 was restricted to areas surrounding the uterine spiral arteries in 14 and 15 weeks samples and increased to the majority of control DSC at 17–19 weeks of gestation (Figure 8C). CD10 was similarly expressed throughout the cytoplasm of control DSC (Figure 8D). In contrast both IGFBP-1 and CD10 levels were significantly reduced, sporadic, or undetectable in the high uterine artery PI SGA decidua (Figures 8C,D).

## Discussion

In summary this paper has shown that PBBx from idiopathic IUGR pregnancies with abnormal uterine artery PI (>1.5) demonstrate a higher number of intact uterine spiral arteries, a failure of both interstitial and endovascular invasion into the decidual myometrial junction, and high numbers of apoptotic mural and interstitial EVT. Numbers of decidual leukocytes were lower in IUGR, while mature CD83^+^ dendritic and CD3^+^ T cells were found in clusters in association with apoptotic EVT in the myometrial portions of these vessels. In the 2nd trimester decidua with high uterine artery PI (>1.6) and SGA fetuses we similarly observed elevated numbers of CD8^+^ T cells and mature CD205^+^ CD83^+^ dendritic cells. Most surprisingly we observed that these high uterine artery PI cases were characterized by increased numbers of lymphatic micro vessels that were packed with decidual leukocytes and a lower expression of markers of mature secretory DSC. Collectively these results provide the first direct evidence that failed progesterone mediated decidualization of the DSCs in early pregnancy leads to (1) persistence of the endometrial lymphatic vessels, (2) a failure of the DSC to recruit the leukocytes into the decidual stroma, and (3) a failure of EVT invasion and developmental maternal immune tolerance in cases of idiopathic IUGR.

Our findings regarding the lack of endovascular EVT invasion in the placental bed biopsies from idiopathic IUGR pregnancies with known abnormal uterine PI suggest that this feature is common with that of pregnancies complicated by hypertension and preeclampsia (3, 5, 15). However, there is some discrepancy regarding the complete lack of interstitial myometrial invasion that we observed in this study. Interstitial EVT were present at low levels in all decidual tissues associated with the IUGR biopsies but appeared to be unable to penetrate the myometrium and many accumulated and fused at the decidual myometrial junction. There is a paucity of studies on PBBx from pure idiopathic IUGR as compared to mixed cases. In a similar study to ours, Lyall et al. contrastingly reported an increase in interstitial EVT density in association with both decidual and myometrial vessels in fetal growth restriction as compared to PE and controls (14). In their study, only 60% of patients in the growth restricted groups displayed a mean uterine artery PI >95th percentile or bilateral notching, while in our study we specifically collected idiopathic IUGR cases with confirmed elevated and abnormal uterine artery PI (>1.5), this may account for the differences between the two studies. In contrast to the pure IUGR cases, the combined IUGR and preeclamptic biopsies in the current study did demonstrate high levels of interstitial invasion into the myometrium suggesting a distinct etiology of pure IUGR. Interestingly EVT from IUGR placenta have been shown to express lower levels of the VCAM-1, alpha 2 beta 1, alpha 3 beta 1, and alpha 5 beta 1 integrin's but much higher levels of intercellular adhesion molecule 3 (ICAM3) than EVT from normal pregnancies (53). The downregulation of these integrin's is likely to negatively affect the ability of the EVT to interact with collagen, laminin and fibronectin, all major extracellular matrix components of the myometrium and decidua, and thus may underlie to inability of the EVT to penetrate the myometrium. Interestingly ICAM3 binds to DCSIGN/CD209+ immature dendritic cells that are found in the decidua, suggesting that it's over expression may lead to changes in the immunological reactions between the mother and the developing fetus and placenta.

During human pregnancy the semi-allogeneic/allogeneic fetal graft is normally accepted by the mother's immune system. Initially the contact between maternal and fetal cells is restricted to the decidua and depends on the reaction between KIR, and NKp receptors on the maternal uNK with paternally-derived non-self-human leukocyte antigens (HLA) class I and class I allotypes (54–56). In our quantitative study decidual leukocytes were shown to be lower in IUGR pregnancies but higher in preeclampsia. We suggest that this is likely to be reflective of a reported decrease in the numbers of uNK in the term IUGR decidua (41). Correlation of this data with the observed increase in the numbers of preserved or partially remodeled decidual vessels, and decreased numbers of enEVT (Supplemental Figure 1B) suggests that this critical interaction of EVT and uNK in early tolerance and decidual vascular transformation is compromised in IUGR.

In the myometrium maternal allo-recognition of the fetus depends on avoidance of maternal T cells recognizing and responding to fetal antigen, due to the restriction of the CD56^+^ uNK to the decidual compartment (57). It is also known that in response to trophoblast-secreted factors, decidual CD209^+^ dendritic cells induce differentiation of CD4^+^CD25^−^ T cells into a CD4^+^CD25^+^FOXP3^+^ Treg population capable of inhibiting CD4^+^ T lymphocyte proliferation (58). In our study we observed apoptotic TUNEL positive mural EVT near maternal leukocyte clusters of macrophage, mature CD83^+^ antigen presenting dendritic cells and CD3^+^ T cells. Recently depletion or malfunction of T cell populations has been associated with recurrent spontaneous abortion and incidence of preeclampsia (59, 60) and higher numbers of CD209^+^ and CD83^+^ DC have been reported in the preeclamptic placental bed (61). Correlation of these studies suggests that the maternal response against the trophoblast may be hostile and excessive in all of these placental pathologies. This is further supported by our quantification of decidual leukocyte populations in the second trimester with high uterine artery PI and SGA fetuses where we found a significant increase in the numbers of cytotoxic CD8^+^ T cells and mature CD83^+^ dendritic cells as compared to the control group. Mature CD83+ dendritic cells are capable of presenting fetal antigen to the adaptive immune cells (62). We have previously shown that in healthy 2nd trimester pregnancy the proportion of CD3^+^ T cells doubles from 1st to 2nd second trimester from 10 to 20% of all CD45^+^ leukocytes in the decidua and is predominated by Th2 CD4^+^ and Th1 CD8^+^ populations, while decidual dendritic cells are maintained in an immature status (27). In the current study the control samples similarly displayed a normal CD4:CD8 ratio (i.e., close to 2 while the high uterine artery PI SGA cases displayed a ratio of 1.21 due to the elevation in CD8+ T cells; this change in T cell ratio is indicative of altered immune function, and chronic inflammation (63). Thus, we suggest that the results in the current study support the hypothesis of activation of the maternal immune system against the fetus and placenta in these cases of maternal idiopathic IUGR. Elevation of CD8 T cells has also previously been reported in the maternal peripheral circulation in cases of IUGR (64). Furthermore, these findings suggest a contributory mechanism to the increased incidence of villitis of unknown origin (VUE), an inflammatory condition of the placenta characterized by maternal T cell infiltrates in the villous stroma in fetal growth restriction and stillbirth (65, 66). In our patient group two of eight IUGR placentas displayed signs of VUE.

The second trimester decidua with high uterine artery PI also provided an unexpected insight into the potential global decidual contribution to the utero-placental pathology of IUGR. It should however be noted that although 4 of 6 cases were shown to be 2 weeks smaller than expected for their gestational age (thus designated as SGA) we cannot conclusively say that these pregnancies, if healthy, would have progressed to IUGR rather than preeclampsia as elevated uterine artery PI has been shown to be predictive for the onset of preeclampsia (49). The most surprising finding in these cases was the persistence of significant numbers of micro-lymphatic podoplanin positive vessels in association with the decidual spiral arterioles. In the basal layer of the non-pregnant endometrium lymphatic vessels are reported to be closely associated with the spiral arterioles (67) and to regress with the onset of perivascular decidualization of the DSC in the late secretory phase such that they are rarely observed in the decidua (68–71). Higher numbers of lymphatic vessels have also been recently found in the decidua of recurrent spontaneous abortions (72), suggestive of a common defect in decidualization in these conditions. In the high uterine artery PI and SGA cases the lymphatic vessels were also packed with decidual leukocytes of uNK, macrophage and T cell lineages, while very few leukocytes were seen in the decidual stroma as is normally observed. Differentiated DSC cells secrete numerous chemokines critical to the recruitment and trafficking of these leukocyte subsets out of the lymphatics and into the stroma, where they play key role in early vascular transformation (21). This again suggested that the decidualization of the DSC was compromised in the high uterine artery PI and SGA cases. Of note, in addition to invading and transforming the uterine arteries EVT have also recently been shown to invade the walls and endothelium of veins and glands and lymphatic vessels in the decidua basalis (72–74). We also observed EVT in venous and lymphatic vessels in both control and high uterine artery PI and SGA cases (Figure 5).

Progesterone is known to play a key role in decidualization of the stromal cells and its effects are primarily mediated via its PR-B receptor. A recent study has shown that in human endometrial stromal cells, PR-B regulates a significantly larger transcriptome than PR-A occupying unique binding sites associated with suppression of cell cycle regulators and induction of angiogenic growth factors and chemokine expression known to be critical in DSC interactions with trophoblast and immune cells (50). In this study we have shown for the first time progesterone responsiveness is decreased in 2nd trimester high uterine artery PI SGA decidua as compared to normal PI controls. The stromal cells of these cases were much smaller and displayed abnormal cellular morphology and far fewer expressed nuclear PR-B and lower levels of the DSC markers IGFBP-1 and CD10. Moreover, PR-B and IGFBP-1 was absent in the most abnormal growth restricted cases expressing SMA in the decidua. The mechanisms underlying PR-B dysregulation are not known however defective menstrual conditioning or epigenetic modulation of PR-B expression has been proposed (75, 76). Interestingly both estrogen and progesterone levels have recently been shown to be lower in the serum of IUGR pregnancies than healthy controls (77), and a recent mouse model of stress induced IUGR has also shown that pregnant dams exhibited reduced progesterone levels and placental heme oxygenase 1 (Hmox1) expression and an increase in cytotoxic CD8+ T cells (78). We therefore conclude, in accordance with the hypothesis proposed by Pijnenborg et al. (30), that progesterone mediated decidualization is negatively affected in cases of high Uterine artery PI and SGA. Interestingly, 7 out of 8 patients in our IUGR PBBx group had either recurrent IUGR or high gravidity but low parity (*G* > 3, *P* < 1) suggestive of a recurrent problem with decidualization, implantation or maintenance of pregnancy.

In conclusion, we have shown that failure of the first step of progesterone mediated DSC decidualization has a fundamental and detrimental effect on all subsequent stages of decidual development in the second trimester. The regression of the decidual lymphatics, migration of the immune cells into the decidual stroma, maternal adaptive immune tolerance, EVT invasion and trophoblast-leukocyte mediated uterovascular transformation were all compromised. Correlation of these findings with our observations in placental bed biopsies supports the hypothesis that defective decidualization leads to the uteroplacental pathology observed in cases of IUGR.

## Data Availability

The datasets generated for this study are available on request to the corresponding author.

## Author Contributions

CD and SL conceptualized the research program and secured the grant funding. CD, CM, SK, JK, and SL conceived and designed the placental bed biopsy study. CD, LH, RJ, and WW conceived and designed the second trimester study. CD, JW, AH, MK, and SW performed the experiments and analyzed the data. CM, WW, and JK recruited and consented patients. CD and CM wrote the manuscript. LH, CD, AH, and CM revised the manuscript. All authors read the manuscript and approved its submission.

### Conflict of Interest Statement

The authors declare that the research was conducted in the absence of any commercial or financial relationships that could be construed as a potential conflict of interest.
