# Supplementary figures and images for: Failure of Decidualization and Maternal Immune Tolerance Underlies Uterovascular Resistance in Intra Uterine Growth Restriction
Source: Front Endocrinol (Lausanne). 2019 Mar 20;10:160. doi: 10.3389/fendo.2019.00160 (PMC6436182; doi:10.3389/fendo.2019.00160)

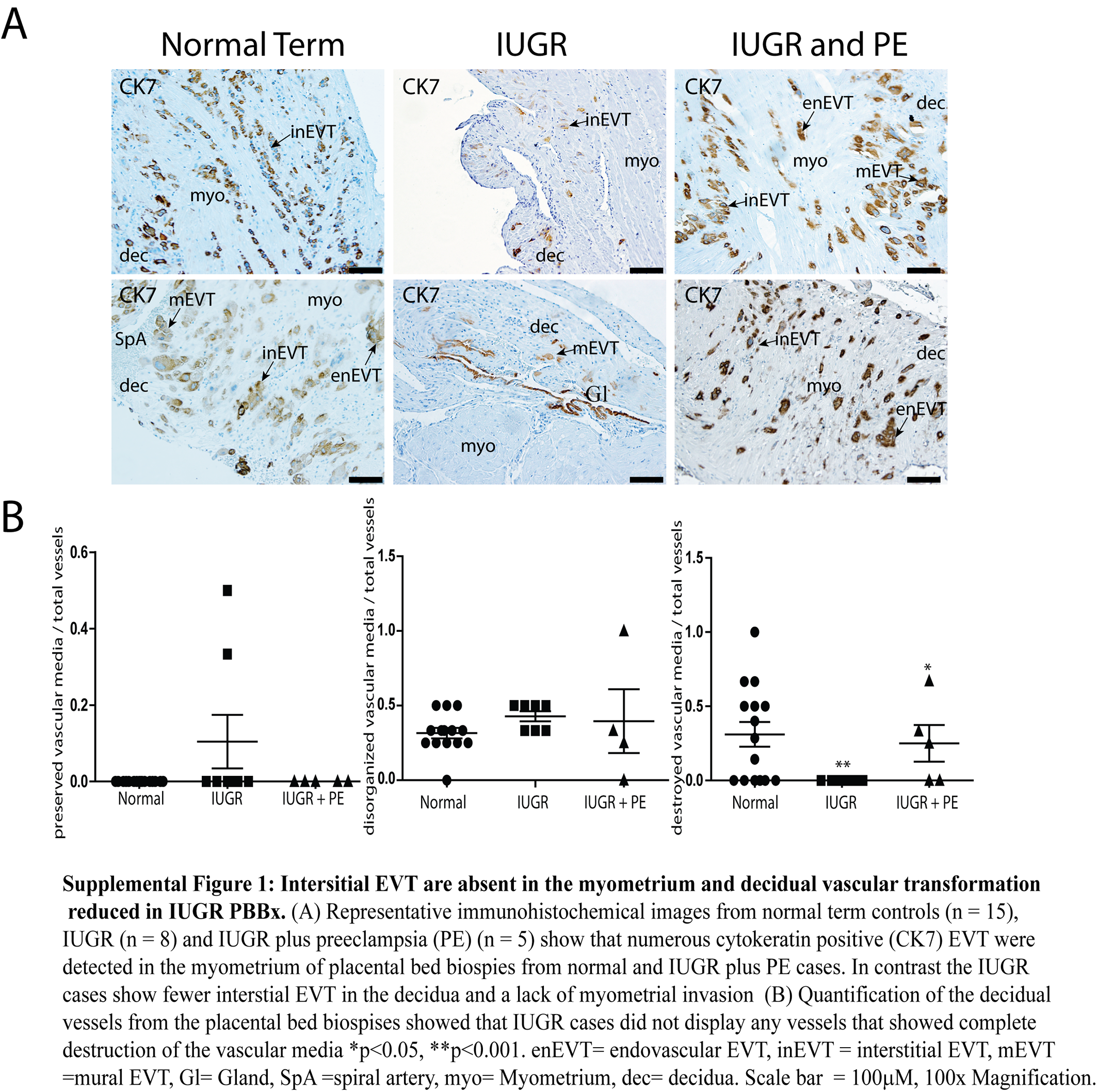

Supplement: Supplementary file 1 [file Image_1.TIF]

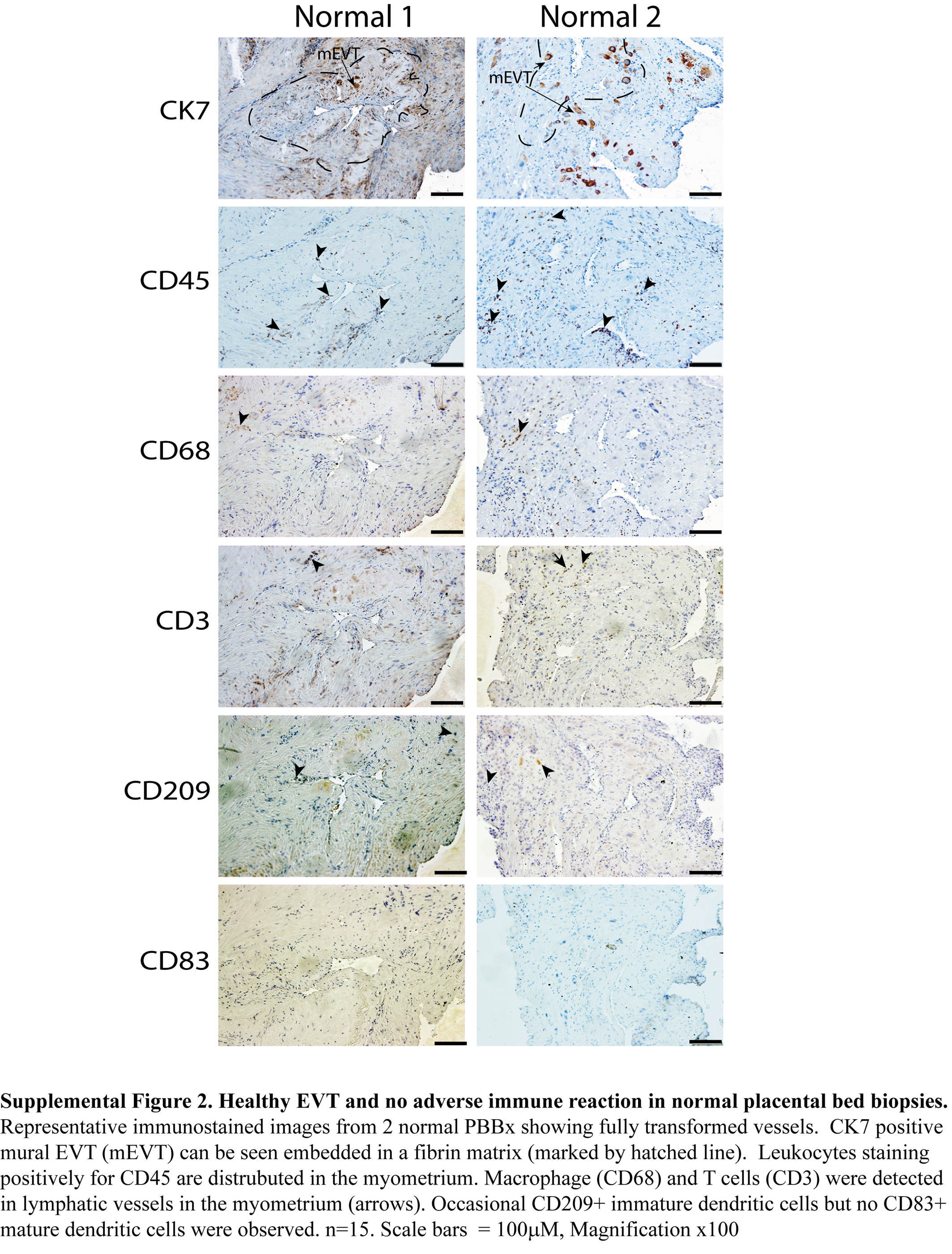

Supplement: Supplementary file 2 [file Image_2.TIF]
